# Population health intervention research: the place of theories

**DOI:** 10.1186/s13063-019-3383-7

**Published:** 2019-06-11

**Authors:** Graham Moore, Linda Cambon, Susan Michie, Pierre Arwidson, Grégory Ninot, Christine Ferron, Louise Potvin, Nadir Kellou, Julie Charlesworth, François Alla, Discussion Panel, François Alla, Pierre Arwidson, Pierre Blaise, Christopher Bonell, Isabelle Boutron, Linda Cambon, Rona Campbell, Patrizia Carrieri, Franck Chauvin, François Dabis, Nancy Edwards, Christine Ferron, Marie-Renée Guevel, Nadir Kellou, Joëlle Kivits, Antony Lacouture, Thierry Lang, Susan Michie, Laëtitia Minary, Graham Moore, Grégory Ninot, Kareen Nour, Jeanine Pommier, Louise Potvin, Lehana Thabane

**Affiliations:** 1 DECIPHer, School of Social Sciences, Cardiff University, Cardiff, UK; 2 Université de Bordeaux, Bordeaux, France; 3 University College London, London, UK; 4 Santé Publique France, Saint-Maurice, France; 5 Université de Montpellier, Montpellier, France; 6 FNES, Saint-Denis, France; 7 Université de Montréal, Montréal, Canada; 8 Université Lyon 1, Lyon, France; 9 A Tree of Life Sciences Ltd, Manchester,, UK

**Keywords:** process evaluation, complex intervention, intervention research

## Abstract

**Background:**

An international workshop on population health intervention research (PHIR) was organized to foster exchanges between experts from different disciplines and different fields. This paper aims to summarize the discussions around some of the issues addressed: (1) the place of theories in PHIR, (2) why theories can be useful, and (3) how to choose and use the most relevant of them in evaluating PHIR.

**Methods:**

The workshop included formal presentations by participants and moderated discussions. An oral synthesis was produced by a rapporteur to validate, through an expert consensus, the key points of the discussion and the recommendations. All discussions were recorded and have been fully transcribed.

**Results:**

The following recommendations were generated through a consensus in the workshop discussions: (i) The evaluation of interventions, like their development, could be improved through better use of theory. (ii) The referenced theory and framework must be clarified. (iii) An intervention theory should be developed by a partnership of researchers and practitioners. (iv) More use of social theory is recommended. (v) Frameworks and a common language are helpful in selecting and communicating a theory. (vi) Better reporting of interventions and theories is needed.

**Conclusion:**

Theory-driven interventions and evaluations are key in PHIR as they facilitate the understanding of mechanisms of change. There are many challenges in developing the most appropriate theories for interventions and evaluations. With the wealth of information now being generated, this subject is of increasing importance at many levels, including for public health policy. It is, therefore, timely to consider how to build on the experiences of many different disciplines to enable the development of better theories and facilitate evidence-based decisions.

## Background

Population health intervention research (PHIR) can be defined as the use of scientific research methods to produce knowledge on policy and intervention programs. Whether or not they are conducted in the context of the health system, these policies and programs have the potential to make an impact at the population level [1]. Population health interventions are generally, but not necessarily, considered as complex interventions, with complexity often seen as arising from their being “made up of various interconnecting parts” [2]. These interventions can also be considered as complex because of the influence of context on their implementation and outcomes [3]. The development of a complex intervention and in particular, the choice of levers targeted depends on explicit or implicit theories about their mechanism of action in their context, an understanding of which can then be enhanced through an evaluation.

The Medical Research Council (MRC) guidance [4] provides recommendations to guide researchers in designing, developing, and evaluating complex health interventions, and more specifically, in evaluating the process [5]. In the overarching MRC guidance, and to a greater extent in the guidance for evaluating the process, there is some emphasis on the use of theory to frame the evaluation: (1) to articulate the causal assumptions behind an intervention and then use this process evaluation frame to understand the implementation, (2) to test mechanisms (and contextual contingencies) and generate emerging insights into the mechanisms, (3) to guide the choice of mechanisms to test, and (4) to understand contextual interactions.

While this guidance represents a key milestone, methods and tools to conduct evaluations need to be refined and there are many outstanding challenges and questions. Notably, the overarching 2008 MRC framework is itself currently undergoing revision, since thinking has moved on significantly in the 11 years since it was published [6, 7]. There is a need not only to develop methods, tools, and practical guidance for researchers, but also to clarify some underlying paradigms and to operationalize the overall research approach, from conceptualization to the dissemination of an intervention.

In France, where PHIR is well developed, the National Coordinated Action for Intervention Research [Action coordonnée pour la recherche interventionnelle en santé publique] (ACRISP) was created in 2015: (1) to support the development of research that is both scientifically sound and useful to practitioners and policy makers, (2) to promote the sharing of experiences between researchers, practitioners, and policy makers, (3) to encourage conceptual and methodological reflections, and (4) to make proposals in terms of organizing research, regulations, and funding.

In November 2016, ACRISP organized an international workshop, bringing together some of the world’s leading experts and researchers. Due to the complexity of the field, which requires an interdisciplinary approach, the objective was to promote exchanges between researchers from different disciplines. The workshop provided an opportunity to share experiences and learning between researchers from various fields, such as clinical research, health services research, and PHIR. The researchers invited were particularly interested in methodological research (most of them had published methodological papers).

Some of the key issues in PHIR were addressed. The presentations and discussions, in three successive sessions, covered various themes. One of them was the place of theory in PHIR. Indeed, according to the MRC guideline, a focal point is to explore the conditions under which an intervention is effective, that is why, for whom, and how does the intervention work? One way to answer these questions as well as possible is to integrate a theoretical reflection into the different steps of an evaluation [8] by: (i) defining the theoretical hypotheses about how the intervention works, (ii) choosing the data to be collected and how they are to be collected (especially for validating the hypotheses), and (iii) defining the transferability conditions, which are the key functions of an intervention, to guide the transfer or the scaling-up of the intervention. Theory could be used within these steps to inform the evaluation of the PHIR.

This article aims to share and synthesize the discussions, works, and recommendations put forward by experts on this subject during the workshop. Practically, the aim is to clarify why and how theories could be better used to improve PHIR, and how to choose and use the most relevant of them in evaluating PHIR. It is not intended to be a systematic synthesis of the science or to present new data, but to be a milestone for a common basis for discussion between researchers from different disciplines and fields.

## Methods

The workshop was organized by GM and FA (who prepared the program and compiled a bibliographic file). It included formal presentations by participants and discussions moderated by GM. At the end of the workshop, an oral synthesis was produced by a rapporteur (PA) to validate, through an expert consensus, the key points of the discussion and the recommendations. All the discussions and the validated synthesis were recorded. The recordings have been fully transcribed. A first draft of the paper was prepared by JC and FA from this material, then corrected and validated by GM, then by all the coauthors (who all participated in the debates).

## Results

### Why use theory in the development and evaluation of PHIR?

The potential focus of a process evaluation for PHIR can be considered as covering everything between the intervention as it is described in a manual and what happens in practice in terms of implementing it. This includes what mechanisms are used, how these processes are shaped, and how they interact with their contexts. Hence, it plays a particularly critical role in developing, testing, and refining theory as part of overarching evaluation studies. One of the objectives of a process evaluation is to understand the mechanisms by which an intervention has had its effect so that more effective interventions can be developed. For complex interventions, it is also necessary to understand which components within a complex intervention are linked with which mechanisms.

Various stages in the development and evaluation of an intervention, including a process evaluation, involve surfacing and interrogating the often latent (nonexplicit) theoretical assumptions regarding how a new set of actions will produce desired outcomes in a particular context, and using a theory of change to frame the questions that a process evaluation needs to pose and what methods will be used to address them. To measure the consistency of intervention delivery with underpinning theoretical principles, it is necessary to start from a point of clarity on what those assumptions are.

Hence, a theory, defined as “a set of analytical principles or statements designed to structure our observation, understanding and explanation of the world” [9], is a useful starting point for developing an intervention, while an evaluation can test and refine the theory. An evaluation to test and refine such theories can maximize their contribution to similar or different contexts and more generally. We agree with the position of various methodological works that a coherent theoretical basis for intervention development and use of evaluations to test key causal assumptions and build theory are crucial [10]. There is a need to be explicit regarding the causal assumptions driving an intervention, whether these are derived from formal social science theory, experience, common sense, or a combination of all of these various forms of “theory” [11].

Nevertheless, to a large extent, the group considered that sometimes people talk at cross-purposes in relation to the various kind of theory. As an illustration, theory has often been taken to mean formal academic theories, called classic theories by Nilsen [9], such as the theory of planned behavior or social cognitive theory. From a realist perspective, all interventions or programs can be viewed as theories [11], because they represent manifestations of assumptions about how an action produces change in a particular context.

So, causes and assumptions always drive interventions and they are all theories, whatever source they are derived from. In practice, interventions in this field draw on quite a wide range of types of theories based on academic research and experience (or common sense), and come from different groups such as researchers and practitioners/actors. Indeed, the outcome of an evaluation can be viewed as being the test of an underlying explicit program theory or the “reconstruction” a posteriori of an implicit theory. Finally, a third case could be the theory of change (ToC) [12] as “a theory of how and why an initiative works which can be empirically tested by measuring indicators for every expected step on the hypothesized causal pathway to impact.” In this case, the theory is different from classic sociological or psychological theories and the middle-range theories of Pawson and Tilley. It is a pragmatic framework used to design and evaluate development programs in many different contexts.

Moreover, using a theory-driven approach could contribute to improving theories [13]. The process of theorizing is always incomplete [14]. Hence, researchers should not treat existing theoretical knowledge as received wisdom and should make the effort to explain what the empirical findings mean for their theory(ies). We underline the necessity (i) to compare the empirical case under investigation and earlier studies that have contributed to the development of the theory(ies) used and (ii) to move beyond simply cataloguing different factors provided by theories and towards an exploration of how these factors work together. The aim of a theory-driven approach is not only to find similarities between the empirical case and extant theory(ies), but also to identify and explain the differences, thus moving the theory(ies) forward.

Hence, clarity about theory, particularly the causes and assumptions, is important for understanding outcome evaluations and implementation fidelity (consistency with function and underlying theory). Interventions delivered in complex systems often look very different, in terms of their precise forms, from one place to another. Therefore, in implementing a good understanding of functions, the causes and assumptions are important for transferability. Clarity about theory is also important for informing future interventions and finding the methods that are likely to be more transferable to other contexts where a problem is due to similar mechanisms, and for understanding which mechanisms work, for whom, and in what contexts. In keeping with this, one concern is to choose the most relevant type of theory to use in an evaluation. Guidance is currently in development for this aspect of uncertainty [15].

### Which theory should be chosen?

The question of which theory to choose is often a dilemma and the subject of much debate. There is a wide range of theories based on academic research and experience (or common sense) and from different groups, such as researchers and practitioners/actors.

For classic theories, the dominant focus tends to be on individual psychology rather than more structural social theories applicable to the population level of the intervention. Indeed, the more individualized nature of intervention theory is commonly acknowledged [16], but there is also a wealth of alternative social science theory for intervention researchers to consider. A more pluralistic approach to the sources of theory could facilitate the development, evaluation, and implementation of interventions that are more effective in addressing PHIR problems [10]. For example, the socioecological approach is increasingly being used in intervention studies that aim to promote healthier behaviors. While socioecological frameworks are typically not explanatory, they provide a framework for drawing together theories from multiple disciplines at multiple system levels, by using social theories or frameworks, such as social determinant frameworks, the theory of diffusion, social networks theories, social capital theories, other professional theories, and organizational theories.

Socioecological theory-based interventions have multiple levels of influence. The individual, with their emotions, knowledge, beliefs, and norms, interacts with a social environment represented by family, friends, and co-workers, all within their living environment (natural or built, and organizational or public policy). So, population health researchers could usefully move towards considering the inclusion of forms of theory that address deeper influences on behavior rather than focusing only on a theory that addresses surface causes. Such changes in theoretical approach are challenging because more complex, system-level theories are not as readily accessible and easy to use as the more simplistic theoretical models. In addition, there is a tendency for recommendations for evaluations to be based on theories in a slightly simplistic way. There has, historically, been a tendency to pick a theory off the shelf (rather than using a bespoke theory of change) and to use it to drive the evaluation. The choice of theory based on conceptual simplicity and because others have used it can lead to the selection of weak theories. Many dominant theories have done little to make interventions more effective [10]. Moreover, evaluation theories are not necessarily organized to be user-friendly and are not necessarily adopted. When a theory is needed, even though there are a lot of intervention theories [17], they are often not used. To avoid this and to choose the best theory, the selection of theories to frame evaluations should be much more based on evidence.

Moreover, much theory is focused on how intervention actions impact health outcomes. However, triggering these mechanisms is contingent on introducing changes to complex systems. There is a need for further holistic approaches that would incorporate a focus on: which existing ways of working will be displaced and how; what new ways of working will be introduced; and how these changes would be expected to impact the target population. In this case, middle-range theories or ToC could accurately be used to hypothesize about and explore these elements. Indeed, these theories actually include elements from classic theories (e.g., motivation theories, Prochaska’s stages of change, etc.) and implementation theories [9] that describe how actions and levers trigger the mechanisms (e.g. self-efficacy, skills, emotional regulation, agentivity, etc.) involved in classic theories.

Therefore, careful consideration of the purpose of using theory is essential, because the purpose should guide the selection of the theory. To guide thinking, to help communicate across disciplines, and to structure research, theories that are less accurate as a representation of reality may be more helpful because they may be easier to work with. However, a beautifully operationalized plan may in some instances count for very little in reality. To really understand and to be able to work with reality and effect change, as well as understanding the complexity of it, we need to be able to drill down to find precision and then scale up to look at the interactions.

### How should theory be used in a PHIR evaluation?

As outlined, evaluation has a role in theorizing and testing intervention mechanisms and also in how interventions interact with their context. There was a consensus among this group that intervention development and evaluation should be driven by theory. Indeed, a theory-driven evaluation [18] could be used to assess the efficacy and consequently the transferability [19] of an intervention.

It may be useful to consider the roles of theory in relation to the MRC guidance. An evaluation of an intervention includes assessing its effectiveness, understanding the change process, and assessing its cost-effectiveness, whilst development includes identifying the evidence base, identifying or developing a theory, and modelling processes and outcomes.

The move towards a greater emphasis on evaluation as a theory-building exercise has led to the notion that the evaluation and development phases can be integrated into the approach for developing and using theory. The development phase has a modelling process and evaluation seeks to understand the change process, which is at the heart of designing and evaluating interventions.

Beyond these frameworks, the group agreed that the key questions relating to theory are as follows:

- Does the outcome fit the theory, i.e., what is the mechanism?
- Can the theory be implemented?
- Can the theory then be used in similar contexts?
- Can the theory be used in other different contexts?

Moreover, we discussed the need in PHIR to take into account different classic theories, among other things, to balance the eco-sociological and behavioral approaches to interventions. We also talked about the particularities of middle-range theories and ToC, and gathered different types of theories that could best be adapted to fit the implementation context. The use of theories in complex interventions needs to take account of many different perspectives and it is important to understand different points of view. For example, investigating social networks may require a combination of epidemiological, psychological, sociological, and health promotion research perspectives, and an interdisciplinary approach may be required. More generally, a process evaluation could be helped by creating an empowering evaluation with public participation. It is very important to involve stakeholders in evaluating a theory because there may already be interventions that seem to be working well, and the aim may be to standardize them, to understand how they work, and to identify the different components needed to understand the intervention, and then to set up a trial or an evaluation of an intervention.

Therefore, the real contribution might be to develop systematic ways of thinking for all disciplines interested in PHIR evaluations, in terms of how to begin to think about the criteria to use and how to think about what theories could be helpful.

### Position statements, further research directions, and recommendations

In these discussions, the experts of the international workshop organized by ACRISP defined six recommendations for using theory in PHIR.

1. Intervention evaluation, like intervention development, should be driven by theory

Exploring why, for whom, and how interventions work leads us to consider the necessity of integrating a theory-driven approach into the different steps of the evaluation: (1) the intervention mapping, which provides explicit hypothetical causal pathways to explore in the evaluation, (2) the choice of data and methods, (3) the definition of transferability, and (4) the scalability of the intervention.

2. Referenced theories and frameworks must be clarified

For classic theories, the use of a published and validated theory or framework should be systematically considered. However, we must distinguish frameworks used in a realistic evaluation or ToC framework where the theory is a combination of (i) academic and evidence-based classic theories, frameworks, and lessons drawn from other experiences and (ii) contextual parameters and stakeholders’ experiences and points of view. Moreover, by offering cases to test theories, the use of a theory-driven approach helps to build a cumulative understanding of the general processes and mechanisms of change, allowing these theories to be refined.

3. PHIR theory should be developed by researchers and practitioners

Theories can be academically driven or developed by the people who design the interventions. The evaluation has to explain the theory, or even reconstruct an implicit theory a posteriori. To do so, the involvement of actors and practitioners is important.

4. More use of social theory is recommended

Social theory should be considered in PHIR to address: (1) changes in social conditions, (2) context, (3) the ways that context shapes behavior, and (4) what happens when programs are unfolded in context. Involving people outside the usual scope needs to be considered, especially multidisciplinary teams in methodological work on how to develop interventions.

5. Frameworks and a common language are helpful in selecting and communicating a theory

Words can have subtle differences that may be really important. Key words need to be defined if they are to be translated to a different context. Using language that is understood by all is key, especially using the same term for the same component, where possible. The most important thing is that everyone knows what is meant. Moreover, transparency is important.

There is potential value in building ontologies with systematic methods for specifying concepts and the relationships between them using a controlled vocabulary framework and taxonomy. Ways of integrating and coordinating different definitions rather than trying to use the same terms and labels may also be useful.

6. Better reporting of interventions and theories is needed

Information on the theory and context is important when the results are as expected, as well as when they are unexpected. For a theory, it is important to know how it was designed in the intervention or the evaluation, or how it was selected, and also how it has been applied.

In developing a theory to guide a program, so that one can evaluate it in terms of the process and mechanisms of action, the important point is to indicate what the concepts are and how they were developed or sourced, and to be transparent about this. In this way, the theory can be evauated in its framework and everybody can learn from it.

Describing things transparently is essential. For example, for complex interventions, descriptions often lack many of the details required to facilitate their replication by others. Journal editors need to implement the guidelines for transparent reporting. Editors and authors should ensure that information on the theory is included and is understandable to the readers and sharable with others, while also taking into consideration both the context and mechanisms.

It may not be possible to describe absolutely everything involved in an intervention, especially a complex intervention, in a journal article, so such details could be provided elsewhere, e.g. protocols, forms, etc. or as appendices rather than summarized.

## Conclusion

Theory has a key place in a process evaluation of PHIR. Theory highlights the role of mechanisms, an understanding of which is essential in the process evaluation.

PHIR should be driven by theory. There are many options for achieving this. The choice of theory and the many different approaches are often a subject of debate. The complexities in this field engender many challenges in developing the most appropriate theories for intervention development and evaluation in specific contexts and also those which can be transferred to similar contexts, or indeed more generally to different contexts.

Nonetheless, there is a wealth of information and experience across many different disciplines, and this subject is of increasing importance at many levels, including for public health policy. It is, therefore, timely to consider how to build on experience from many different disciplines to enable the development of better theories and facilitate evidence-based decisions.

The consensus reached by this group is that theory-driven intervention and theory-driven evaluation are key in PHIR. The group has provided some current thinking and suggestions to take this forward.
